# Contrast-enhanced ultrasound (CEUS) in abdominal intervention

**DOI:** 10.1007/s00261-018-1473-8

**Published:** 2018-02-15

**Authors:** Dean Y. Huang, Gibran T. Yusuf, Mohammad Daneshi, Raymond Ramnarine, Annamaria Deganello, Maria E. Sellars, Paul S. Sidhu

**Affiliations:** Department of Radiology, King’s College Hospital, Denmark Hill, London, SE5 9RS UK

**Keywords:** Ultrasound contrast agents, Ultrasound, Intervention, Contrast enhanced ultrasound, Abdominal intervention, Image guided intervention, Interventional radiology

## Abstract

The introduction of ultrasound contrast agents has rendered contrast-enhanced ultrasound (CEUS) a valuable complementary technique to address clinically significant problems. This pictorial review describes the use of CEUS guidance in abdominal intervention and illustrates such application for a range of clinical indications. Clinical application of CEUS discussed include commonly performed abdominal interventional procedures, such as biopsy, drainage, nephrostomy, biliary intervention, abdominal tumor ablation and its subsequent monitoring, and imaging of vascular complications following abdominal intervention. The purpose of this article is to further familiarize readers with the application of CEUS, particularly its specific strength over alternative imaging modalities, in abdominal intervention.

Image guidance improves the safety and effectiveness of abdominal intervention and creates opportunities for minimal invasive therapies [1–5]. Ultrasound (US), fluoroscopy, and computed tomography (CT) are commonly used for image-guided procedures. US confers many distinct benefits [6]: dynamic multiplanar imaging with continuous real-time visualization of needle placement and target, repeatability with absence of ionizing radiation, good patient tolerability, and portability with the potential to be performed at any location in a variety of clinical settings. However, the limitation of lack of enhancement information with conventional US, in comparison with other enhanced imaging modalities such as CT, remains a major technical factor which hinders success of US-guided procedures [7]. Targets such as abdominal masses and complex intra-abdominal collections may be isoechoic to the background and, therefore, being poorly differentiated from adjacent structures. Procedures initially considered for US guidance may ultimately be performed with CT guidance due to the difficulty with target visualization [8]. When a procedure initially attempted with US is unsuccessful and requires an alternative imaging modality, it can result in higher costs, increased patient anxiety and discomfort, and potentially prolonged procedure time and logistical scheduling conflicts [9].

With the introduction of microbubble ultrasound contrast agents (UCAs), the issue of lack of enhancement with conventional US was addressed. UCA have been available in most parts of the world for more than 20 years. More recently, Lumason (Bracco Diagnostics; Monroe Township, NJ) has been granted FDA approval in the United States for abdominal applications for investigation of focal hepatic lesions in both the adult and pediatric populations [10]. There is also accumulative experience on the use of UCA for a variety of wider clinical indications including US-guided interventions, culminating in a number of published guidelines [11, 12].

Performance with contrast-enhanced ultrasound (CEUS)-guided abdominal intervention, as in other image-guided procedures, depends not only on the operator’s technical skill, but also on the knowledge embedded in the imaging technology, available tools, and existing protocols [13]. The purpose of this article is to consider the literature on CEUS-guided abdominal intervention, and to exhibit representative cases where CEUS proved valuable, to further familiarize readers with this technique. The specific strength of CEUS over conventional US and alternative imaging such as CT and fluoroscopy in abdominal intervention is also summarized in Table 1.

**Table 1:** Summary of CEUS applications for various abdominal interventions

Abdominal interventions	Specific strengths of CEUS over conventional US	Specific strengths over enhanced CT or fluoroscopic-guided intervention
Drainage	Ability to differentiate avascular complex abscesses from other vascular masses Improve delineation of abscess boundary and surrounding enhancing parenchyma Improve depiction of the avascular necrotic portion, loculation and internal septation Minimize the risk of inadvertent injury to the adjacent organ Endocavitary CEUS through the drainage catheter facilitates monitoring of the position and patency of catheters within collections Endocavitary CEUS allows visualization of communication between a collection and adjacent structures	Multiplanar imaging which allows approach from different angles Repeatability without risk of iodinated contrast-related nephrotoxicity Portability with potential to be performed at any location Real-time imaging of abnormal communication with adjacent structures with absence of ionizing radiation
Biopsy	Demonstration vascularization to enable differentiation between isoechoic tumor and normal parenchyma Improve biopsy-positive yield by revealing the vascular portion of the target Provide spatial information of necrotic areas to avoid Improves visualization of atrophic parenchyma in renal biopsy for evaluation of nephropathies	Multiplanar imaging which allows approach from different angles Negate need for nephrotoxic contrast medium or ionizing radiation Portability with potential to be performed at any location
Percutaneous nephrostomy insertion	Intravenous CEUS improves the visibility of the non-vascularized renal calices Improve success for puncturing non-dilated systems Endocavitary CEUS can verify correct placement of nephrostomy tube Confirm drainage prior to removal of nephrostomy	No risk of distracting blob of contrast material that interferes with the procedure if initial attempt fails Negate need for ionizing radiation Portability with potential to be performed at any location
Biliary intervention	CEUS cholangiography can confirm biliary drainage catheter is positioned adequately Improve visualization of the biliary system particularly in non-dilated biliary system Capacity to depict complications associated with the trans-hepatic drainage, such as an arterial connection with a percutaneous biliary drainage tube	Negate need for ionizing radiation Improved temporal and spatial resolution in imaging complication such as arterial fistula Portability with potential to be performed at any location including patient’s bedside
Thermal ablation of abdominal tumors	Allow lesions inconspicuous on conventional US to be visualized Allow assessment of ablation zone for residual disease immediately post-ablation to determine if a repeat ablation is required Allow assessment of vascularity of ablation scar in followup period	Allows multiple approach planes Procedure guidance in real-time Repeat assessment of ablation zone vascularity immediately post-ablation and during surveillance without need for ionizing radiation burden or use of nephrotoxic iodinated contrast medium
Gastrointestinal application	Endocavitary CEUS allow assessment for complication such as a leak due to dislodged gastrostomy tube	Negate need for ionizing radiation Portability with potential to be performed at any location including patient’s bedside
Detection of vascular complication	Active hemorrhage and pseudoaneurysm could be positively identified on CEUS	Capability to scan the region of interest continuously, thus excluding the theoretical risk of missing a delayed extravasation Can image pseudoaneurysm and confirm success of embolization while patient is on the operating table Negate need for ionizing radiation or nephrotoxic contrast medium during follow up
Pediatric application	Allow enhanced vascular and endocavitary US examination in pediatric population to aid intervention	Negates the need for ionizing radiation or general anesthesia required for alternative therapeutic approach

## Ultrasound contrast agents

Microbubbles in the UCAs consist of an inert filling gas, such as sulfur hexafluoride, encapsulated by phospholipid shells [14]. Microbubbles resonate with low acoustic power and oscillate in a nonlinear fashion, and the harmonic signals produced can be selectively detected on US systems with special multipulse CEUS-specific software [15]. The biocompatible shells of microbubbles are metabolized by the liver, and filling gas exhaled by the lungs. UCA are not nephrotoxic, thus dispensing of any laboratory renal function tests prior to intravenous administration. Contraindications for the administration of UCA are few, namely, known allergic reaction to the UCA, severe pulmonary hypertension, and pregnancy [16]. Despite the low risk, resuscitation equipment should be accessible and trained personnel should be available for adverse events.

## CEUS with intravascular administration of UCA

Microbubbles in UCA remain exclusively intravascular when administered intravenously, making them ideal for assessment of both micro- and macro vasculatures. Visualization of the vascularity of an abnormality offered by CEUS conveys its use to image-guided intervention through improved localization of a focal abnormality. Conversely, lack of perfusion can be established conclusively in avascular abnormalities such as abscesses.

CEUS should be performed after an unenhanced conventional US examination is fully evaluated. This allows the operator to identify the area of interest, and establish suitability of a subsequent CEUS examination. UCA are most commonly manually injected as a bolus through an intravenous line, usually via an antecubital vein, followed by 10 mL of 0.9% normal saline flush. It is generally best to insert the intravenous line after the baseline conventional US examination to avoid unnecessary cannulation in case a CEUS examination is not deemed worthwhile. However, due to logistical reasons, patients who are likely to benefit from intravenous UCA administration may need placement of an intravenous line prior to conventional US study at many facilities. The volume of UCA administered varies depending on the structure investigated, sensitivity of ultrasound machines and the UCA used. Conventionally, intravenous use of UCA requires 1.2–4.8 mL depending on the site examined. A dose of 2.4 mL of Lumason per injection is considered adequate for the liver and other abdominal procedures. After administration by intravenous injection, UCA lasts for about 4–5 min in the circulation. A second dose of equal volume can be administered if required.

## CEUS with endocavitary administration of UCA

Endocavitary CEUS is a developing technique [17, 18]. Manual injection of air through agitated saline solution has been utilized in many endocavitary ultrasound applications [19, 20] as the technique is inexpensive and readily available. Ultrasonography contrast media for endocavitary application has, however, evolved with new microbubble UCA with improved contrast media stabilization and small but sufficiently echogenic microbubbles, resulting in superior image quality. It is currently recommended that endocavitary CEUS could be considered if alternative imaging methods carry a higher risk for the patient, e.g., need to transport critically ill patients [11]. Broadly speaking, clinical indications for endocavitary CEUS include confirmation of drain placement and effective drainage, evaluation of a physiological or non-physiological cavity, and detection of communication between two cavities through a fistula tract. The use of endocavitary CEUS in the biliary system [21], urinary collecting system [22], and “hysterosalpingo–contrast sonography” [23] has been evaluated in clinical studies.

Endocavitary CEUS differs from intravenous CEUS, in that the volume of the solvent is much smaller than the full blood circulation volume, so a much smaller dose of UCA is required. Endocavitary CEUS can be performed by first diluting UCA in 0.9% normal saline, and the resulting solution is then injected into tubes or cavities. Adequate dilution of UCA is essential to avoid posterior acoustic shadow artifacts secondary to a high concentration of microbubbles. In our experience, this could be achieved with a dilution of 0.1 mL Lumason in approximately 40–50 mL of 0.9% saline. The administered volume of the solution with diluted UCA varies on a case-by-case basis dependent on the estimated volume of the cavity. The lack of circulation of UCA in a confined cavity means microbubbles remains stable within a collection for up to 20–30 min [24]. Destruction of microbubbles by a high-energy ultrasound pulse is, therefore, necessary before repeating an endocavitary CEUS evaluation. No adverse reactions relating to endocavitary CEUS have been documented in a limited number of clinical studies. Nevertheless, contraindications for intravenous administration of UCA should be taken into consideration when performing an endocavitary CEUS.

## CEUS applications in abdominal intervention

### Drainage

**Fig. 1:**
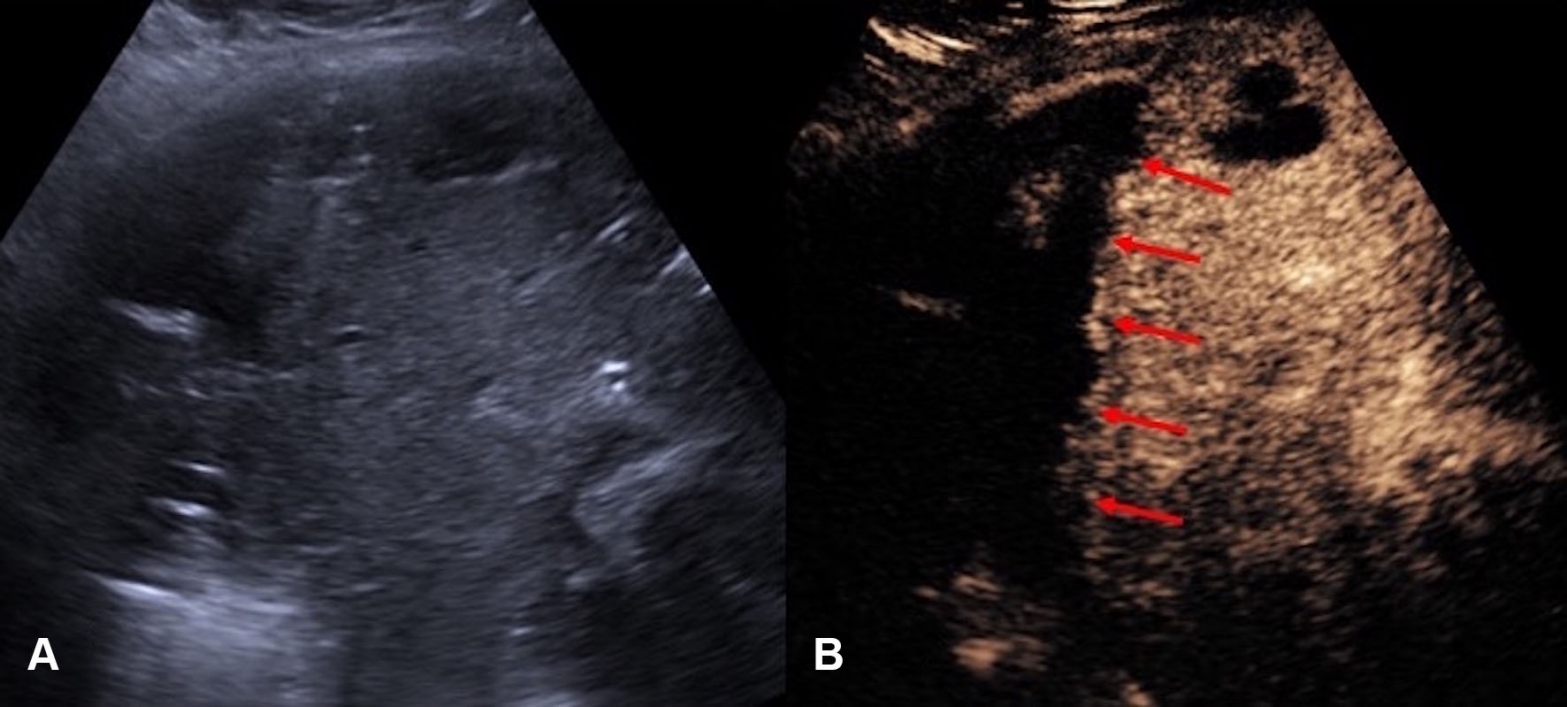
**A** Grayscale US. **B** CEUS of a hepatic abscess. Grayscale US image shows the abscess as a heterogeneous echogenic area with poorly defined margin underestimating the true extent of abscess. CEUS enables better delineation of the liver abscess and reveals a sharp boundary (red arrows) between the collection and hepatic parenchyma

**Fig. 2:**
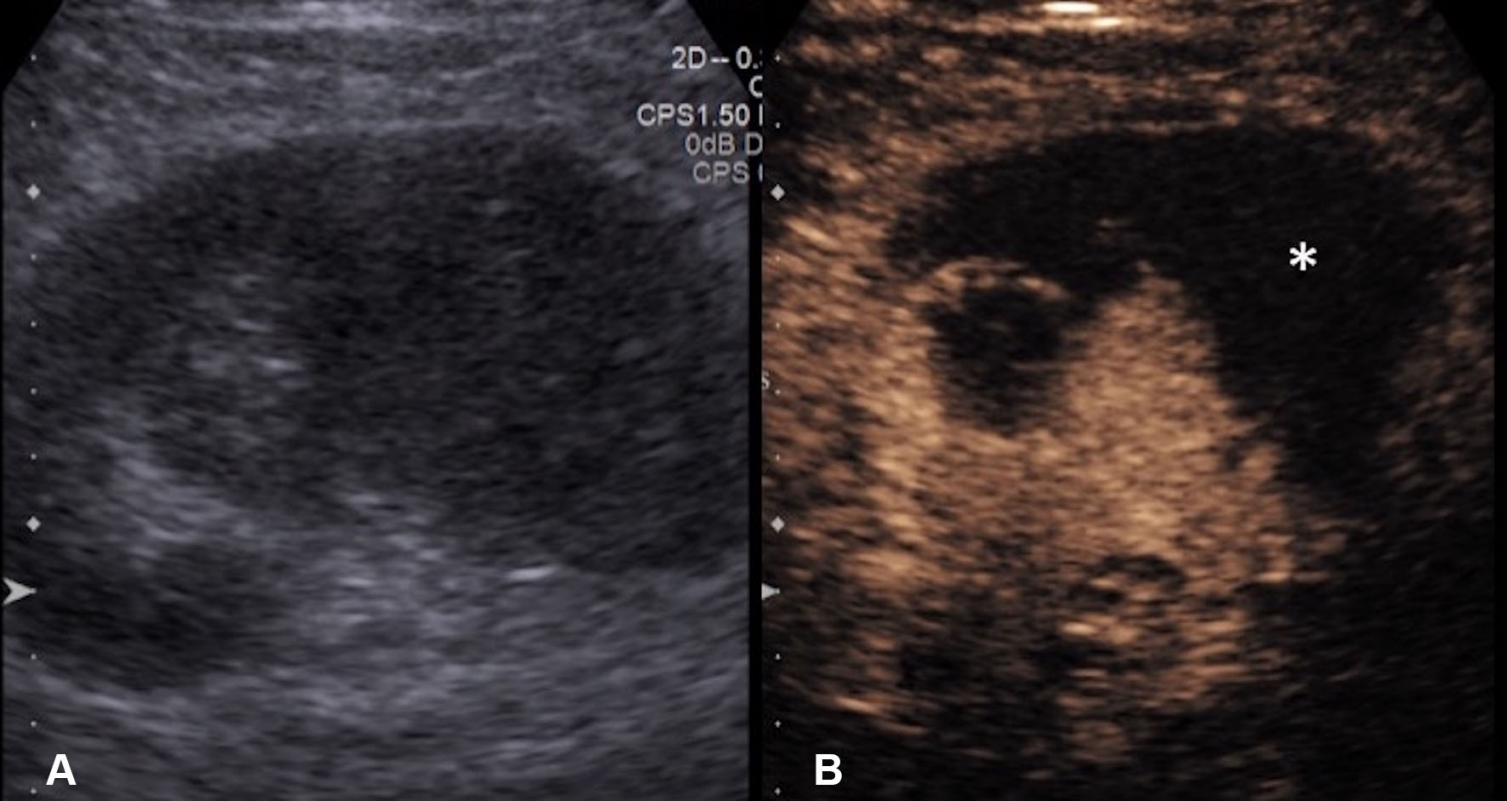
**A** Grayscale US. **B** CEUS of a sub-capsular renal abscess. The boundary of the non-enhancing sub-capsular collection (asterisk) is clearly shown on the CEUS but not on the grayscale US image. CEUS guidance allows insertion of the drainage catheter to be performed confidently and reduces the risk of damaging the adjacent renal parenchyma

US and CT-guided percutaneous drainage is routinely used as the treatment for a variety of intra-abdominal collections. While sterile fluid collections can be easily identified on gray-scale US, complex collections such as hepatic abscesses, pancreatitis-associated fluid collections, retroperitoneal, and bowel-related abscesses, may appear almost solid and mass-like. The variation in sonographic appearances of complex intra-abdominal collections may make accurate diagnosis difficult due to the resemblance in their sonographic features to those of other pathology [25]. CEUS facilitates the identification of abscesses through demonstration of lack of vascularity within them [26]. CEUS enables better delineation of liver abscess and reveals a sharp boundary between the collection and surrounding hepatic parenchyma [27] (Fig. 1). An abscess can be more precisely targeted under CEUS guidance, with augmented depiction of the avascular portion and internal septation, allowing adequate placement of a drainage catheter. Moreover, if a collection is in close proximity to an abdominal organ, CEUS could outline the unenhanced collection precisely and minimize the risk of inadvertent injury to the adjacent organ (Fig. 2).

**Fig. 3:**
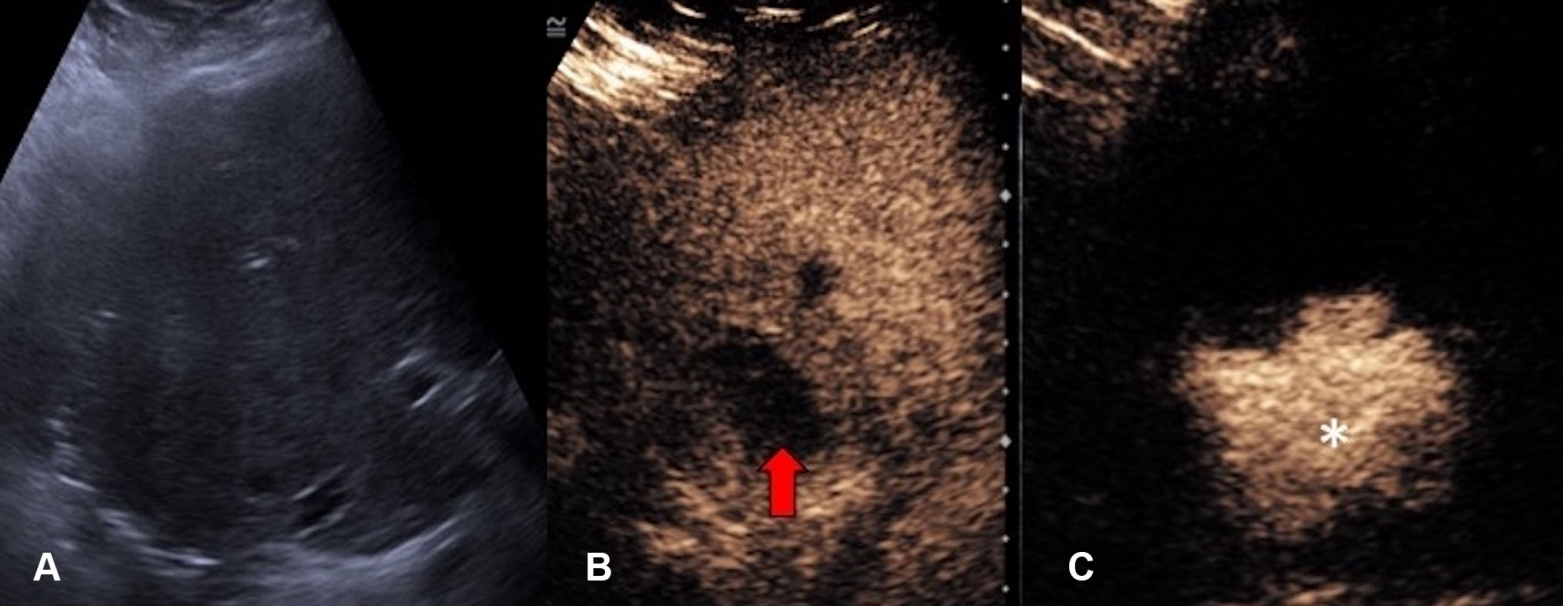
**A** Grayscale US. **B** Intravascular CEUS and **C** endocavitary CEUS of a hepatic abscess. Intravascular CEUS shows the enhancing hepatic parenchyma surrounding the non-enhancing abscess (red block arrow), whereas endocavitary CEUS, with the UCA instilled through the drainage tube, confirms adequate drainage catheter position and shows the morphology of the abscess cavity (asterisk)

**Fig. 4:**
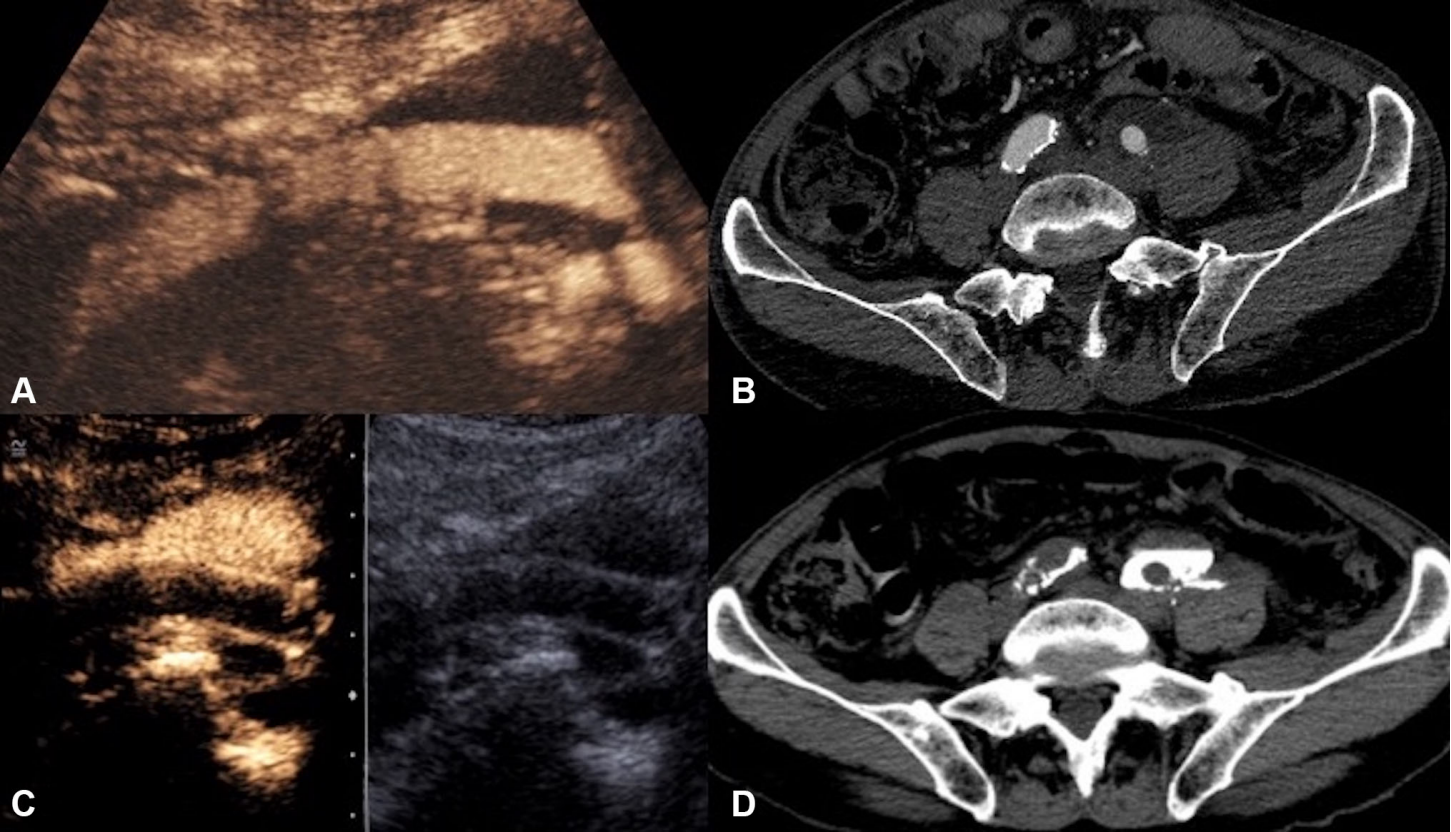
**A** Intravenous CEUS. **B** Arterial phase image of a contrast-enhanced CT. **C** Endocavitary CEUS with UCA injected through the nephrostomy. **D** Urographic phase image of a contrast-enhanced CT of a patient with ureteric injury and urinoma formation following a vascular bypass surgery. CEUS studies (**A**, **C**) definitively excluded communication between the fluid collection and the left iliac vascular graft. Although the subsequent CT (**B**, **D**) demonstrates similar findings, CEUS provided real-time imaging and instant diagnosis at patient’s bedside

Endocavitary CEUS with injection of UCA through a drainage catheter can be performed either immediately after drainage catheter insertion or in the subsequent follow-up period. Tubes and drains are easily detected on gray-scale US but their tip positions are sometimes difficult to locate. Endocavitary CEUS through a drainage catheter primarily facilitates confirmation of the catheter position within the collections. It also provides additional assessment of effectiveness of drainage, particularly in a collection with multiple loculations [17] (Fig. 3). Furthermore, when the clinical scenario requires an answer to the question on whether any communication between a collection and an adjacent structure exists, endocavitary CEUS through a drainage tube may prove to be valuable as conventional US would be unlikely to provide sufficient information. The excellent temporal and special resolution of endocavitary CEUS allows real-time demonstration of a communication between compartments through visualization of the movement of, even a small quantity, microbubbles, without need for further alternative imaging such as contrast-enhanced fluoroscopy or CT studies (Fig. 4).

## Biopsy

**Fig. 5:**
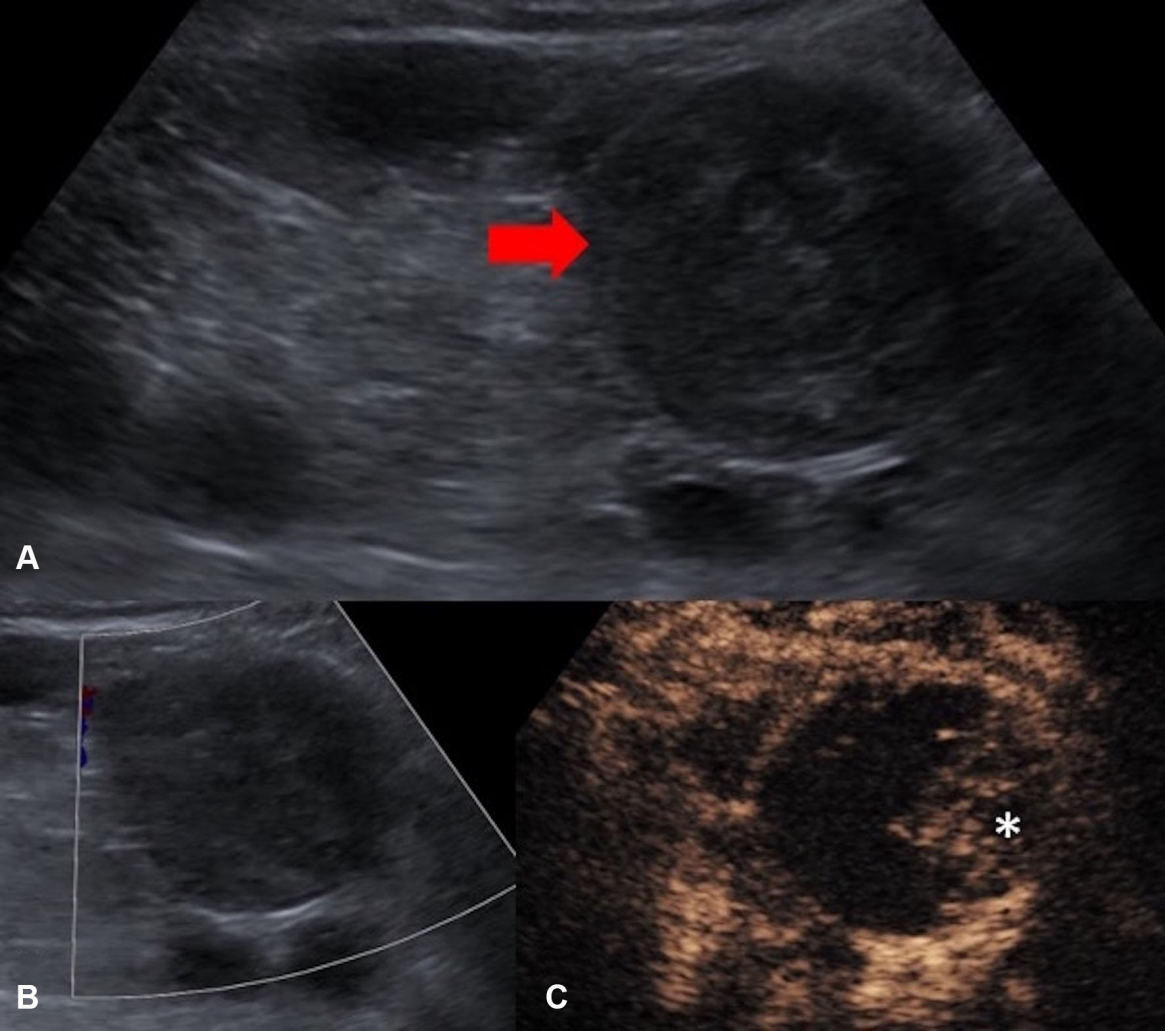
**A** Grayscale US. **B** Color Doppler US. **C** Intravenous CEUS of a large renal cell carcinoma with central necrosis (red block arrow). The grayscale and color Doppler US do not show differentiation between vascular and avascular portions of the tumor. CEUS clearly shows the avascular necrotic portion (asterisk) of the tumor to be avoided during biopsy

**Fig. 6:**
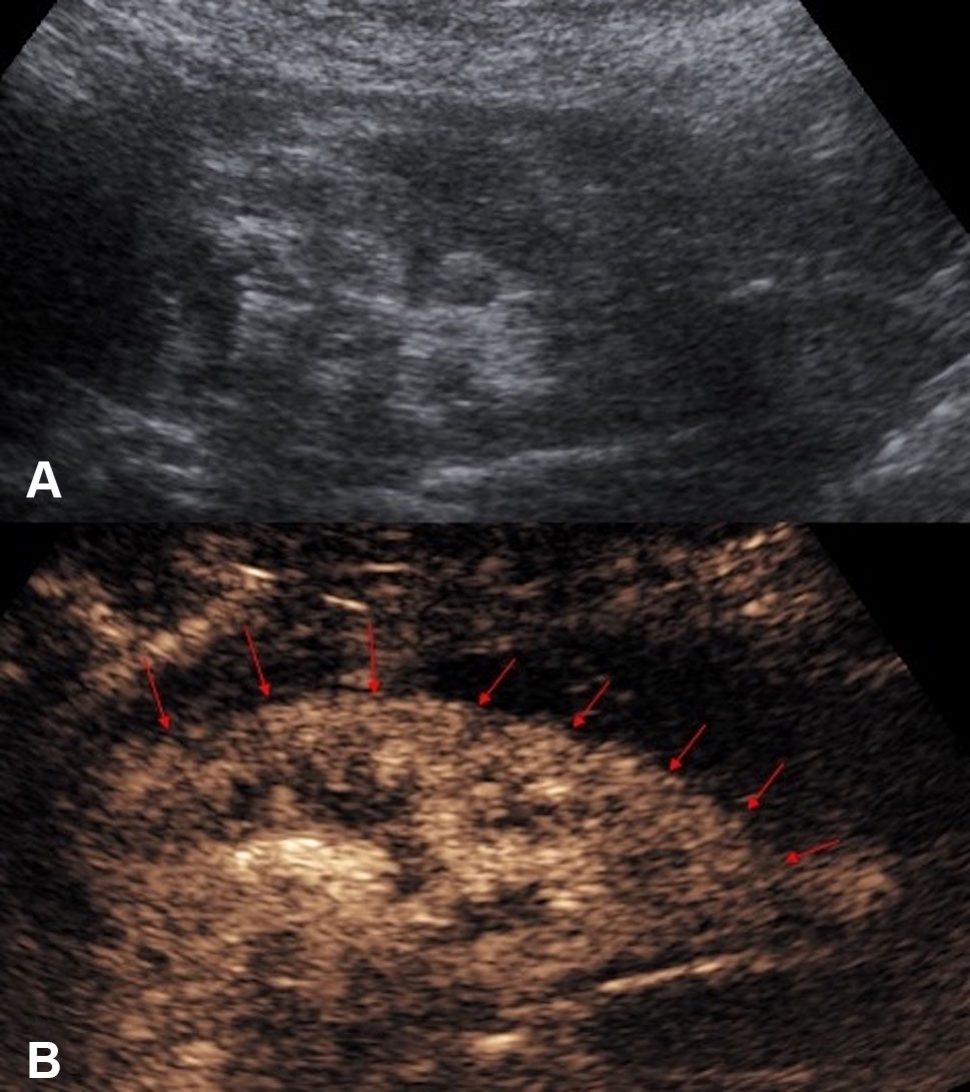
**A** Grayscale US. **B** CEUS of an atrophic kidney targeted for a non-focal biopsy for the sampling of the renal cortex for evaluation of nephropathies. CEUS guidance improves visualization of the atrophic kidney by differentiating the enhancing renal parenchyma from the background tissue, with better delineation of the enhancing renal parenchymal outline (red arrows)

One of the main issue to be confronted in image-guided percutaneous biopsy procedures is the lack of imaging differentiation between the target and adjacent structures [7]. Poor differentiation of the target lesions from adjacent tissue is a common reason for failed US-guided procedures [28]. Not infrequently, a focal lesion is visible only on post-contrast diagnostic CT and cannot be adequately discerned during an US-guided biopsy procedure. CEUS is well placed to solve this problem because of its capacity to differentiate between the altered vascularization of a tumor and surrounding parenchyma [10]. CEUS guidance has been widely applied during biopsies in liver, kidneys, and other abdominal locations [29–32]. CEUS offers the potential to improve positive biopsy yield by revealing the vascularized, potentially viable or more active, portion of a lesion [33]. CEUS guidance can also be advantageous in providing spatial information of necrotic areas to avoid during biopsy, such as in biopsy of a large tumor with central necrosis [34] (Fig. 5). Furthermore, sampling of atrophic, often echogenic, renal cortex of kidneys in patients with renal insufficiency for evaluation for nephropathies [35] could be better assisted with CEUS guidance, as small, but enhancing, kidneys are better visualized on CEUS than on gray-scale US (Fig. 6).

## Percutaneous nephrostomy and nephrostogram

**Fig. 7:**
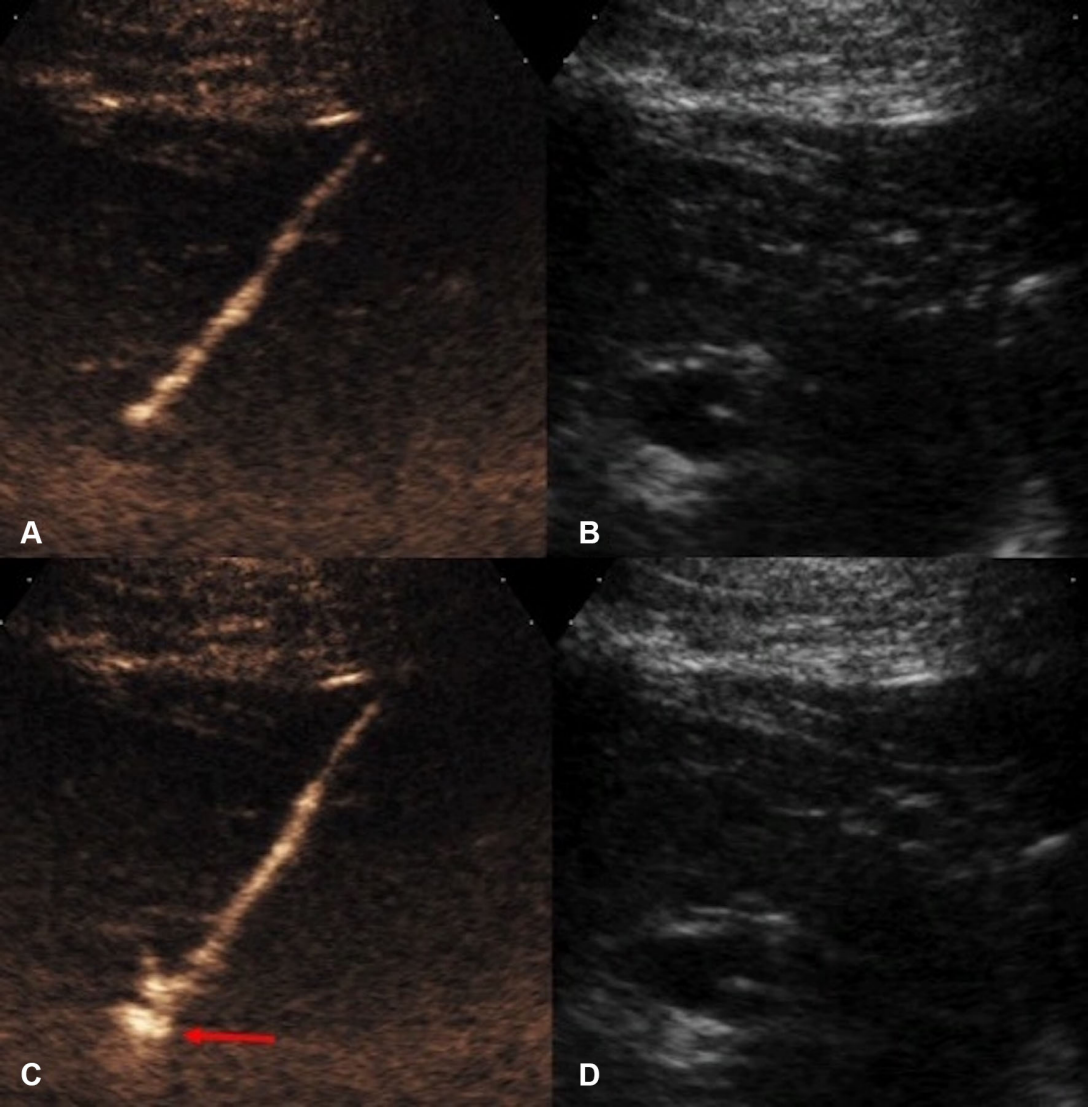
CEUS-guided nephrostomy puncture. **A** CEUS and **B** grayscale images before access into the collecting system was obtained. The stylet of the access needle for nephrostomy puncture was removed and the lumen of the needle was pre-filled with a small drop of diluted microbubble UCA. **C** CEUS and **D** grayscale US images obtained immediately after the collecting system is punctured. Microbubbles are visualized in the renal collecting system (red arrow) the instance the successful puncture is made

US-guided percutaneous nephrostomy (PCN) is routinely used in patients with clinical necessity of urinary drainage or urinary diversion. Frequent technical difficulties contributing to a failed PCN include lack of visibility of a target calyx [36] in cases of non-dilated collecting systems or when echogenic blood clots, pus, or debris is present within the renal calices. Intravenous CEUS improves the visibility of the calices by revealing the non-vascularized renal pelvicaliceal system against the background enhancing renal parenchyma [33]. In addition, specific techniques for CEUS-assisted puncture for PCN, with administration a small volume of diluted UCA through the puncture needle, have been described [33, 37]. With these techniques, a successful puncture can be instantly confirmed either when microbubbles reflux back along the needle due to back pressure of urine, or when microbubbles are visualized in the renal collecting system (Fig. 7). CEUS-assisted PCN offers a problem-solving adjunct in challenging cases including in non-dilated systems, showing high success rate and acceptable complications [36]. When compared to conventional fluoroscopic guidance, CEUS guidance offers a further technical advantage that if the initial placement is inaccurate, microbubbles can be destroyed and, therefore, would not leave a distracting blob of contrast material that interferes with the procedure, as in the cases of procedures performed with iodinated contrast material.

**Fig. 8:**
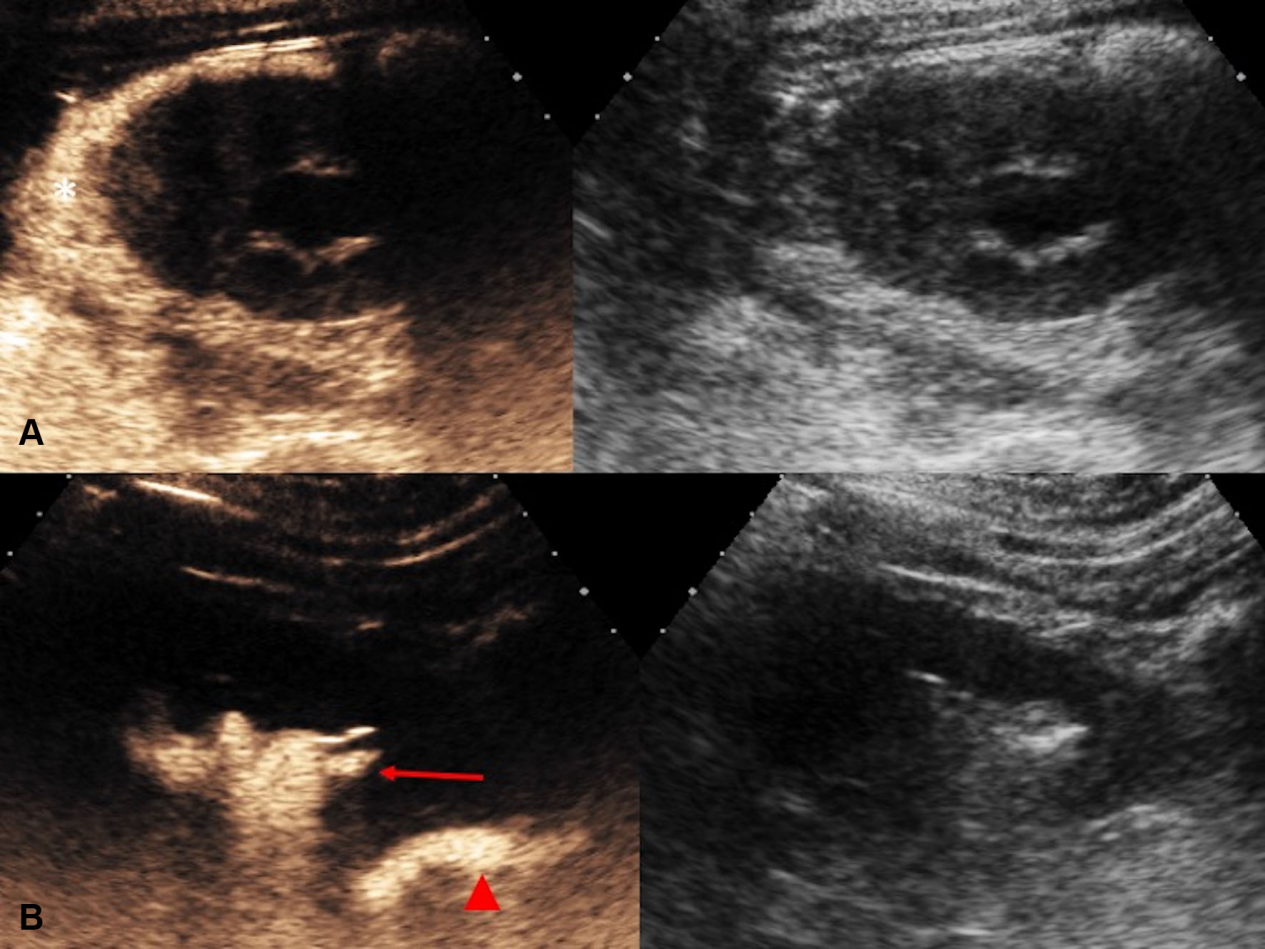
Endocavitary CEUS (left) and co-registered grayscale US (right) nephrostography images. UCA can be injected through the drainage catheter to verify the correct placement of the nephrostomy tube. In row (**A**), the nephrostomy tube is dislodged and there is pooling of microbubble contrast in perinephric spaces (asterisk). CEUS nephrostography following re-insertion of the nephrostomy tube (row **B**) confirms adequate nephrostomy placement with visualization of microbubble contrast in the renal collecting system (red arrow) and proximal ureter (red arrow head)

**Fig. 9:**
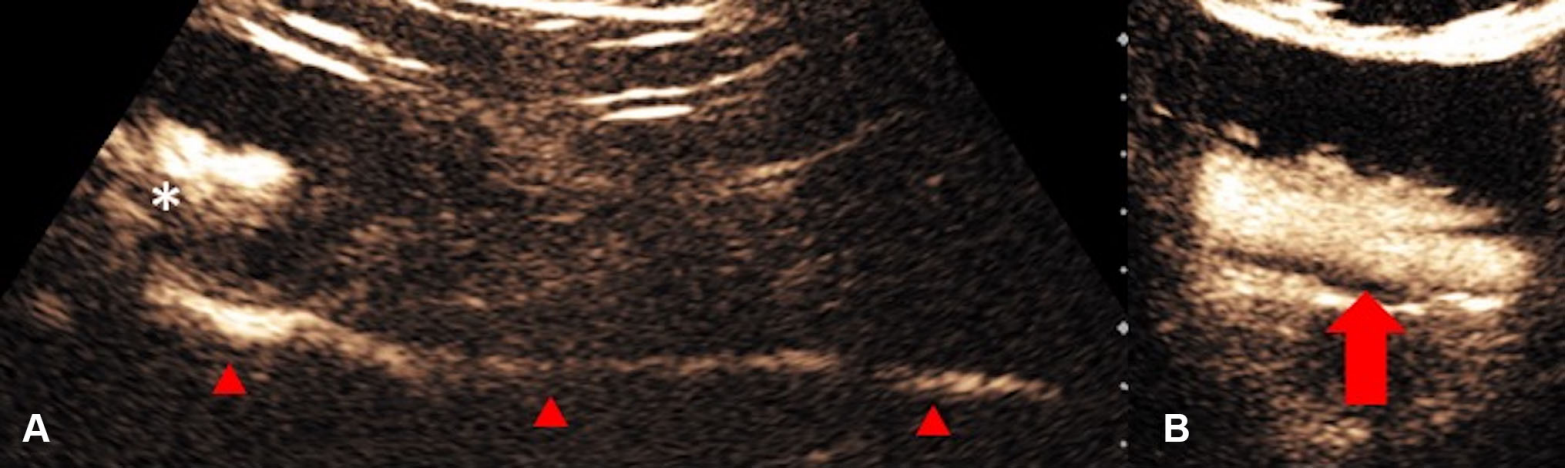
CEUS nephrostography. Following a successful nephrostomy insertion, diagnostic evaluation can be obtained with a CEUS nephrostography by introducing microbubbles into the collecting system. **A** The renal pelvic (asterisk) and the ureter (red arrow heads) can be visualized. **B** Patent drainage into the bladder can be confirmed by the presence of microbubbles (red block arrow) in the bladder

Following a successful PCN, it is frequently deemed necessary to assess the renal collecting system and ureter for free passage of urine prior to nephrostomy removal. The existing experience in CEUS voiding urosonography has showed safety of administration of endocavitary UCA within the urinary tract without side effects [22]. UCA can be injected through nephrostomy catheters to verify the correct placement (Fig. 8). Unobstructed drainage into the bladder can also be confirmed with a CEUS nephrostography with visualization of microbubbles in the bladder (Fig. 9). The degree of spatial resolution of endocavitary CEUS nephrostography gives a high degree of confidence of free ureteric drainage and a recent study shows 100% concordance with fluoroscopy [38]. CEUS nephrostography thus has the potential to become a feasible alternative to conventional fluoroscopic nephrostography [39]. CEUS nephrostography is particularly suitable in patients with contraindications to iodinated contrast material, or for ill patients as it can be performed at bedside.

## Biliary intervention

**Fig. 10:**
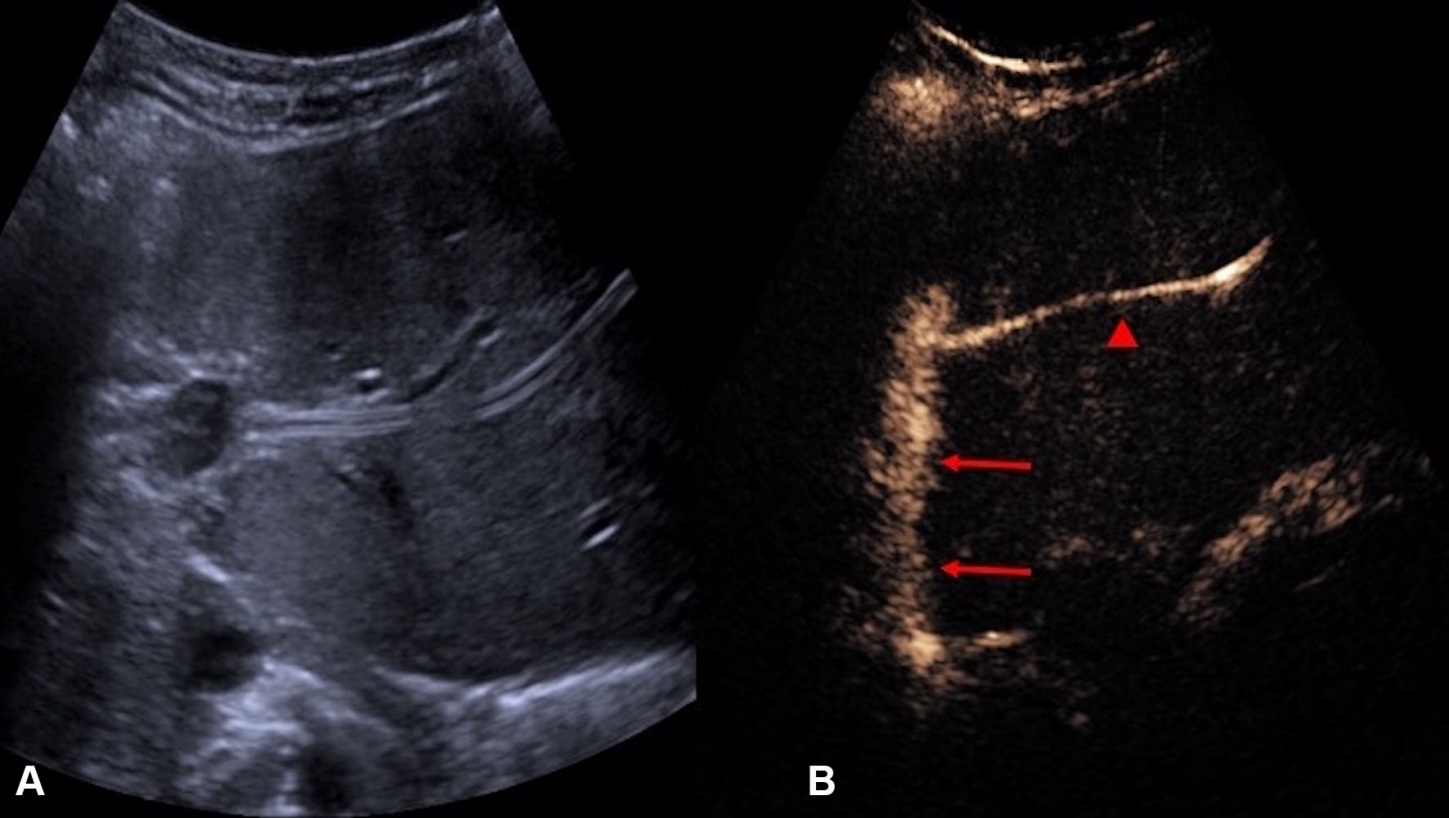
**A** Grayscale US and **B** endocavitary CEUS cholangiography: Endocavitary CEUS cholangiography with administration of UCA into the biliary system through drainage catheter confirms adequate placement and patency of the biliary drain (red arrow head) and drainage into the hepaticojejunostomy (red arrows)

**Fig. 11:**
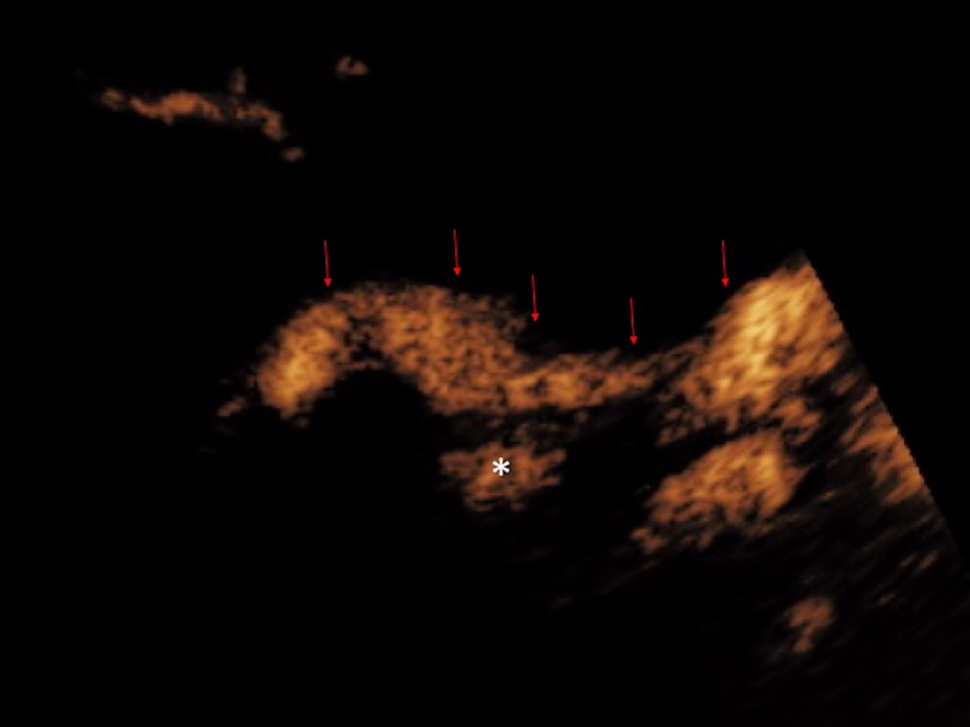
Endocavitary CEUS cholangiography: Endocavitary CEUS cholangiography with administration of UCA into the biliary system through a drainage catheter demonstrates drainage through the biliary anastomosis into the hepaticojejunostomy (red arrows). Pooling of microbubble contrast (asterisk) is noted near the anastomotic region, raising the suspicion of presence of a small biliary leak. However, caution should be exercised in interpretation of this finding, as pooling of microbubble contrast within a cystic duct remnant may display a similar appearance

Percutaneous trans-hepatic biliary drainage is commonly performed for the treatment of biliary obstruction. It is critical to determine whether the biliary drainage catheter is positioned adequately to ensure the effectiveness of the drainage. The position of the tip of a biliary drain is often difficult to visualize on conventional US due to bowel gas. A fluoroscopic cholangiography is often required following percutaneous biliary drain insertion or a biliary stent placement. In patients with contraindication to iodinated contrast material or in children in whom ionizing radiation exposure is undesirable, CEUS cholangiography has the potential to offer a practical alternative. CEUS percutaneous trans-hepatic cholangiography was first described in 2009 [40]. Percutaneous access to biliary system can be assisted with CEUS with intravenous administration of UCA to better depict the biliary system, particularly in cases where the biliary system is non-dilated [41]. Endocavitary CEUS with administration of UCA into the biliary system through drainage catheter enables determination of the adequacy of the drainage catheter position [41]. In addition, studies evaluating CEUS cholangiography have shown favorable results in delineating the anatomy of the bile duct tree and confirming bile drainage or the level of impediment to the drainage of bile [21, 42–44] (Fig. 10). It has also been reported that CEUS cholangiography can reveal complications associated with the percutaneous trans-hepatic biliary drainage, such as an arterial communication with a percutaneous biliary drainage tube [45], due to its superior temporal and spatial resolution. Furthermore, evaluation of post-surgical complications such as a bile leakage can be performed with CEUS (Fig. 11) but the accuracy for this may be limited on occasion by the presence of bowel gas [41] or pooling of microbubble contrast within a cystic duct remnant. Despite this, CEUS cholangiography following percutaneous drainage could be an advantageous alternative for the assessment of the function of a biliary drain over conventional fluoroscopy in critically ill patients by allowing bedside examination [11].

## Thermal ablation of abdominal tumors

With the advent of thermal ablation technologies, percutaneous ablation has emerged as a viable treatment option as an alternative to surgery in the management of solid abdominal tumors. Frequently utilized ablative mechanisms include radiofrequency ablation (RFA), microwave ablation (MV), cryoablation, particularly for hepatocellular carcinoma [46, 47], hepatic colorectal metastases [48] and renal tumors [49].

**Fig. 12:**
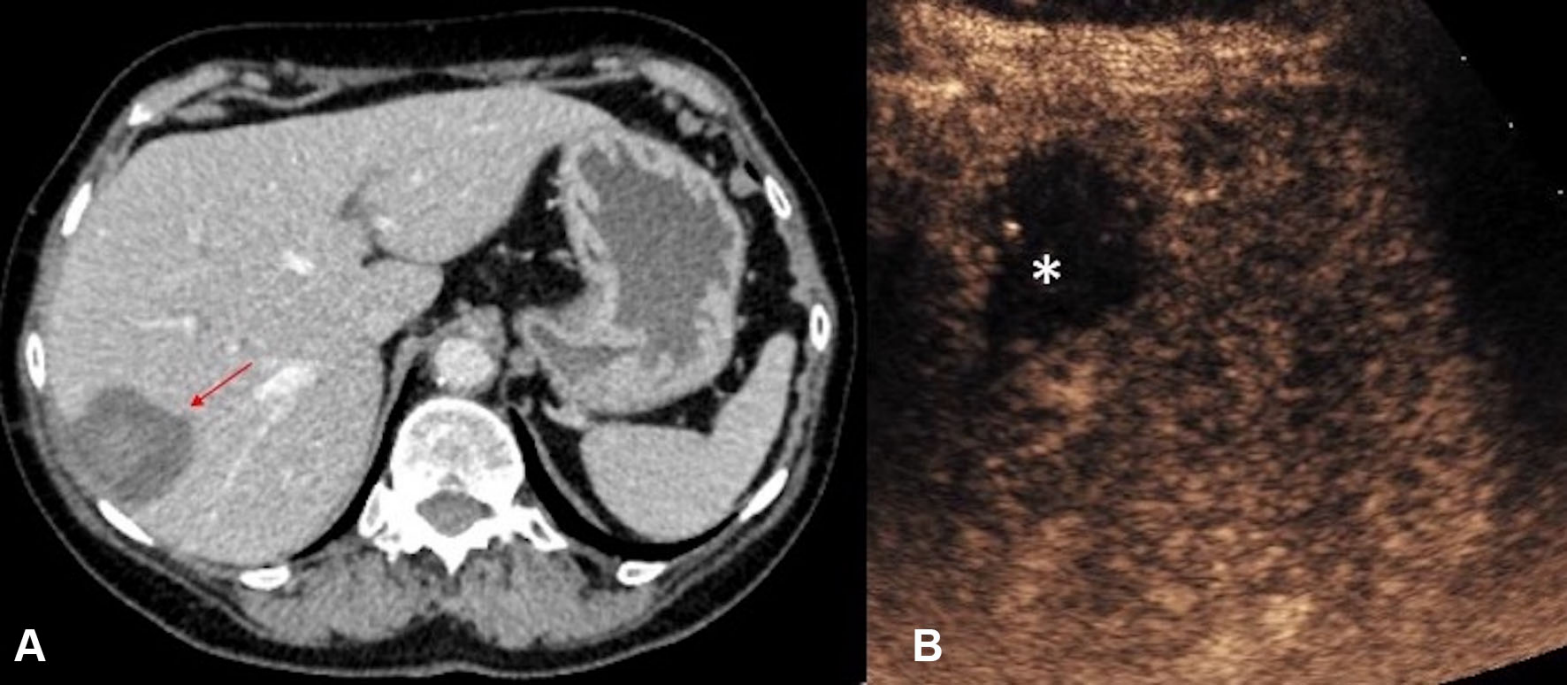
**A** CT (red arrow indicates the ablation zone) and **B** CEUS images obtained in the immediate post-ablation period following a microwave ablation of a small hepatocellular carcinoma. With its improved temporal and spatial resolution for micro-vascularity, CEUS permits exclusion of any residual vascularity at the ablation zone (asterisk) with confidence

Percutaneous ablation of solid abdominal tumors involves the placement of ablation probes into the tumor masses under image guidance. Accurate intra-procedure delineation of tumors and post-ablation evaluation of the ablation zone are fundamental to the effectiveness of treatment. Conventionally, procedural guidance can be achieved with CT or US. US allows multiple planes to perform the procedure in real time. However, even in the most experienced hands, some lesions are poorly visualized on gray-scale US and there can be difficulty in differentiating viable tumors from areas of normal parenchyma or necrosis [50]. CEUS facilitates the treatment as it provides enhancement information that allows lesions inconspicuous on conventional US to be demonstrated during ablation procedures in real time [51]. CEUS also allows improved visualization of residual disease either intra-operatively or immediate post-procedure. With its improved resolution for micro-vascularity, US performed with UCA permits definitive exclusion of any residual vascularity post-ablation [52] (Fig. 12). Abnormal nodular hypervascular region at the peripheral ablation zone on CEUS can be regarded as residual viable tumor [53], and potentially be treated in the same setting [54]. However, in this regard, operators should be mindful of the presence of confounding periablation hyperemia or gas bubbles at the ablation site, which could bring challenges to the interpretation of CEUS appearances immediately post-ablation [55]. Periablation hyperemia often demonstrates a uniform rim of enhancement which, unlike residual tumor, persists throughout the different enhancement phases. Gas bubbles at the ablation site are markedly echogenic on gray-scale US and can be recognized prior to the start of CEUS exam.

### Thermal ablation in liver

Percutaneous thermal ablation therapy is a widely used method for liver tumors, which has been a curative method for small liver cancer treatment [46]. Sonographic guidance is a feasible technique but hepatic tumors can be difficult to be visualized on conventional US, especially after trans-catheter arterial chemo-embolization (TACE). One study [56] reported that tumors could not be visualized on grayscale US in 30% of the patients referred for percutaneous RFA. CEUS has been extensively utilized in thermal ablation procedures for liver tumors that are undetectable via US [57, 58] and evaluation of the ablation zone in the immediately post-procedure [59]. In addition, CEUS using Sonazoid (Daiichi-Sankyo co., Tokyo, Japan) as a UCA in guiding a procedure such as biopsy or RF ablation of liver lesions has been described. Sonazoid microbubbles are taken up by Kupffer cells in the reticuloendothelial system of the liver, with malignant lesions which contain few or no Kupffer cells clearly shown as contrast defects in Kupffer-phase imaging to facilitate intervention [60].

### Thermal ablation in kidneys

Recent advances in nephron-sparing procedures have evolved the management of renal tumors. Ablative technologies offer an alternative nephron-sparing treatment to surgery for small renal tumors [61]. Invisibility of renal tumors on conventional US remains the main limiting factor for performing thermal ablation of small renal masses under US guidance [62, 63]. Because of the low resolution of the gray-scale and the low sensitivity for smaller arteries and arterioles, some small isoechoic renal tumors cannot be visualized on conventional US, especially in the deep sections of the renal medulla [64]. The usefulness of CEUS in the assessment of renal anatomy, renal vascularity and focal renal tumors, as well as the assessment of percutaneous ablation therapies has been highlighted in published CEUS guidelines [11, 12]. Due to the excellent imaging of micro-vessels with CEUS, the isoechoic, small renal cell carcinoma and complex residual tumors, even hypo-vascular tumors, can be identified [65]. CEUS was utilized for the guidance percutaneous ablation of renal cell carcinoma in several reports with positive clinical results achieved [66–69]. Note should be made that CEUS cannot provide information about the excretory function or exclude collecting system injury, as UCA are purely intravascular when administered intravenously.

### Post-ablation monitoring

**Fig. 13:**
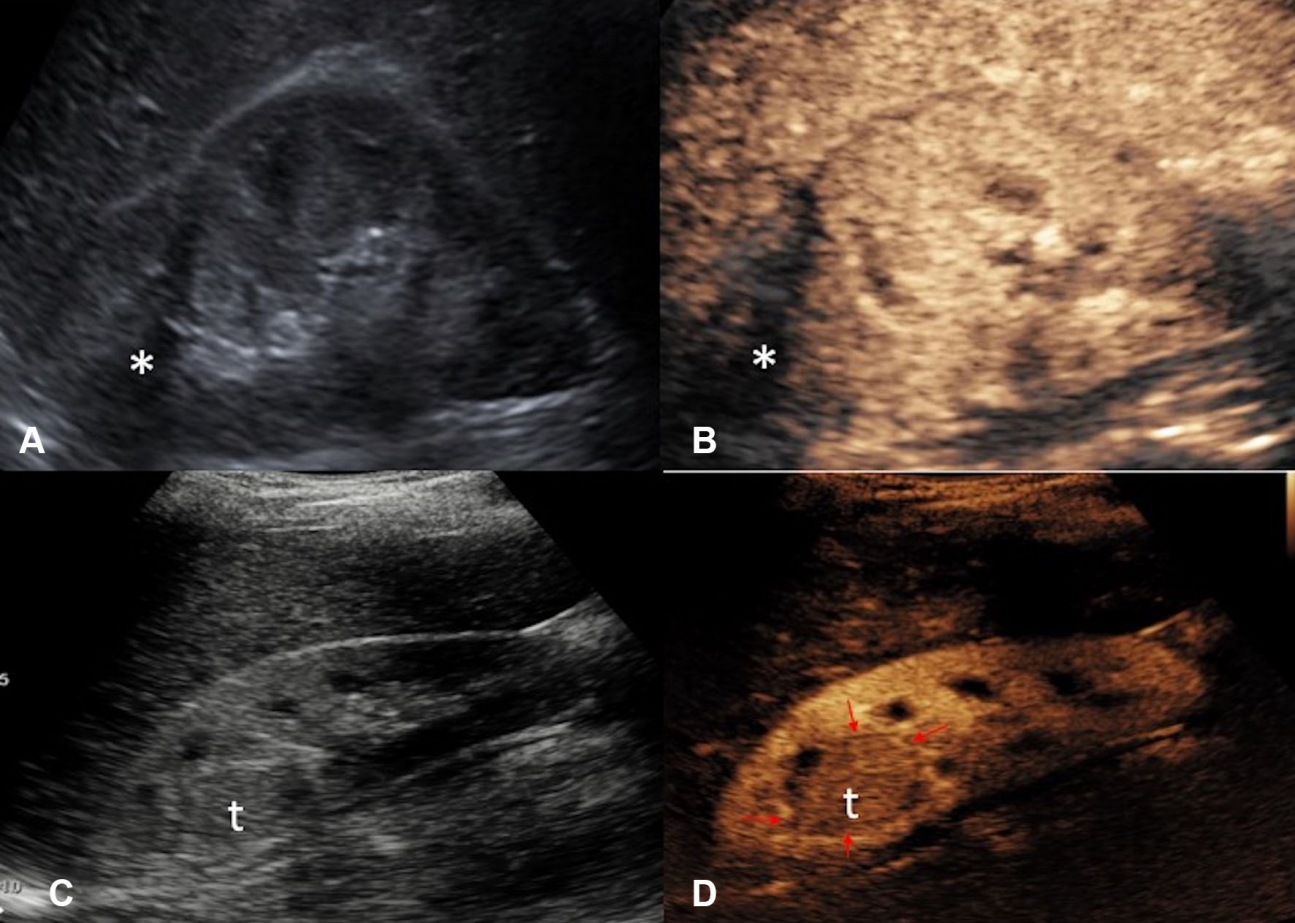
**A** Grayscale US and **B** CEUS images of the right kidney 6 months followup after cryoablation of an upper pole renal cell carcinoma. The cryoablation scar (asterisk) is noted but there are no features suggestive of local recurrence. **C** Grayscale US and **D** CEUS images 3 years following cryoablation of the same kidney with recurrence at the ablation site. The recurrence at the ablation scar (*t*) is of a similar echo reflectivity to the surrounding normal renal parenchyma on grayscale US. The margin (red arrows) of the isoechoic recurrence of renal tumor (*t*) is better delineated with CEUS due to the excellent ability of CEUS to image the differential vascularity between the tumor and surrounding renal parenchyma

During surveillance following ablation therapy, the ablation scar may be of a similar texture to the surrounding normal tissue on grayscale US. The clarity in assessment of tissue vascularity achieved with CEUS could be used for monitoring effectiveness of ablation therapy and detecting local disease recurrence (Fig. 13). It compares favorably with CT and MRI in follow-up assessment of tumors that were treated with ablation therapy [69–71]. CEUS surveillance thus offers an alternative to reduce radiation burden and nephrotoxic contrast medium load for patients in surveillance following tumor ablation therapy.

### Gastrointestinal application

**Fig. 14:**
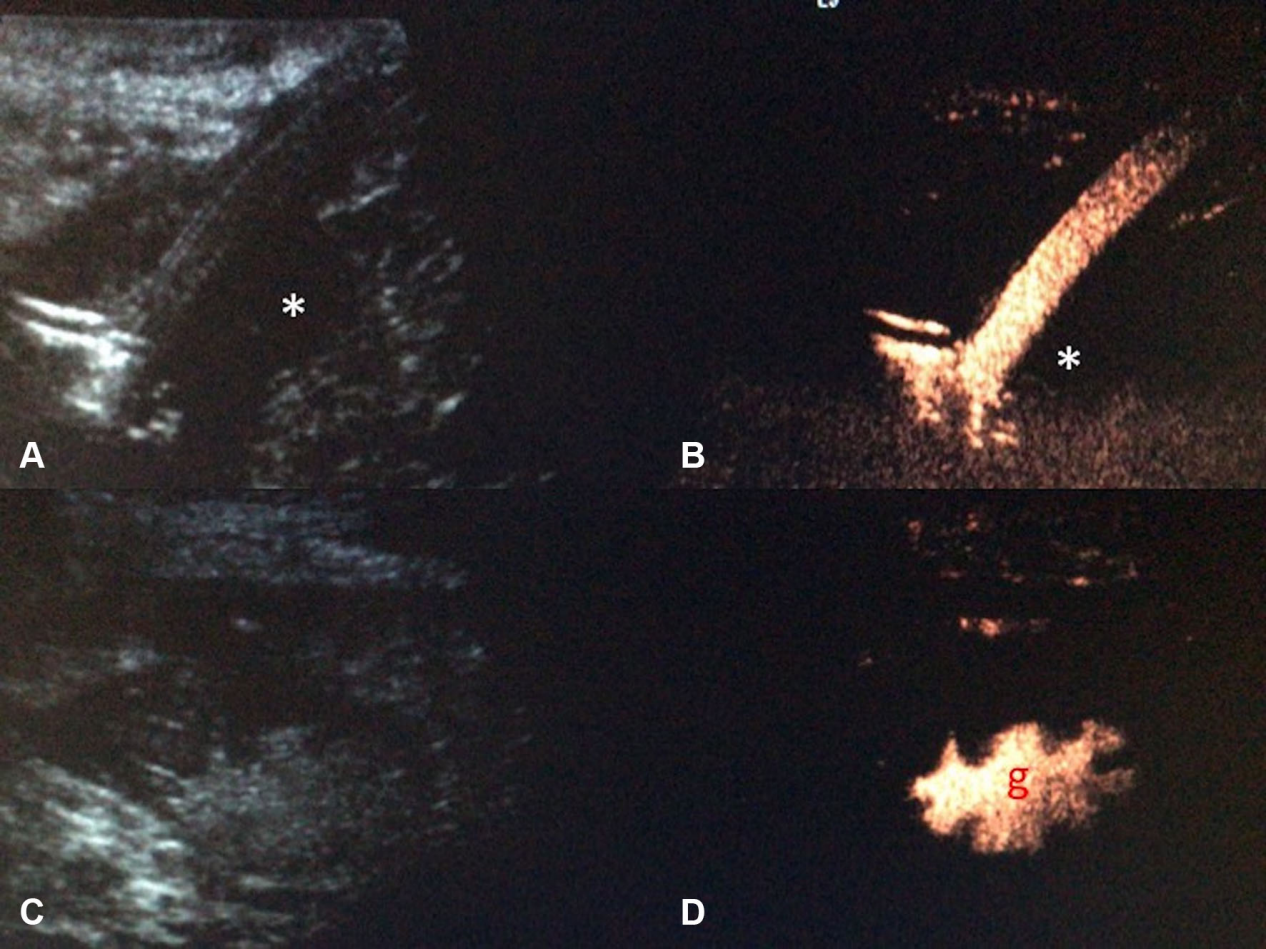
Grayscale US and endocavitary CEUS images of a radiologically inserted gastrostomy (RIG) tube. A small hypoechoic collection (asterisk) is present in grayscale US (**A**). Endocavitary CEUS (**B**), with administration of microbubble contrast through the gastrostomy tube, showed no accumulation of microbubble contrast in the collection, thus excluding an ongoing leak. Correct placement of the gastrostomy tube is further confirmed with visualization of microbubble contrast on CEUS (**D** with the corresponding grayscale US (**C**)) within the gastric cavity (*g*), which is recognizable due to presence of gastric rugae

Endocavitary CEUS offers potential in detecting complications relating to abdominal intervention such as radiologically inserted gastrostomy tubes [72]. UCA can be administered via a gastrostomy tube to confirm correct placement, and exclude presence of a leak (Fig. 14). CEUS could be performed with real-time imaging, and in a variety of clinical settings for instant diagnosis.

## Detection of vascular complication following abdominal intervention

**Fig. 15:**
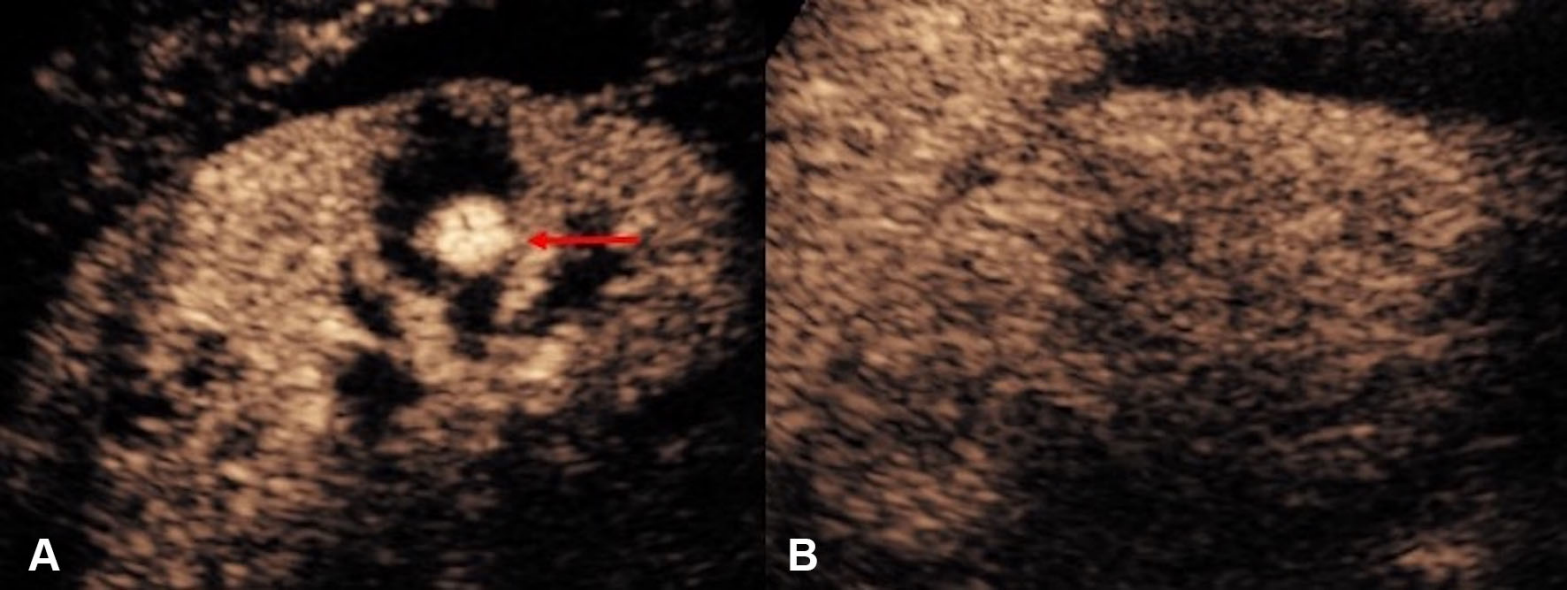
Post-renal biopsy pseudoaneurysm. **A** CEUS demonstrates a pseudoaneurysm (red arrow) within the right kidney. **B** CEUS performed 1 week following embolization of the pseudoaneurysm demonstrates absence of the pseudoaneurysm and normal perfusion of the surrounding renal parenchyma, confirming the success of the selective embolization procedure

**Fig. 16:**
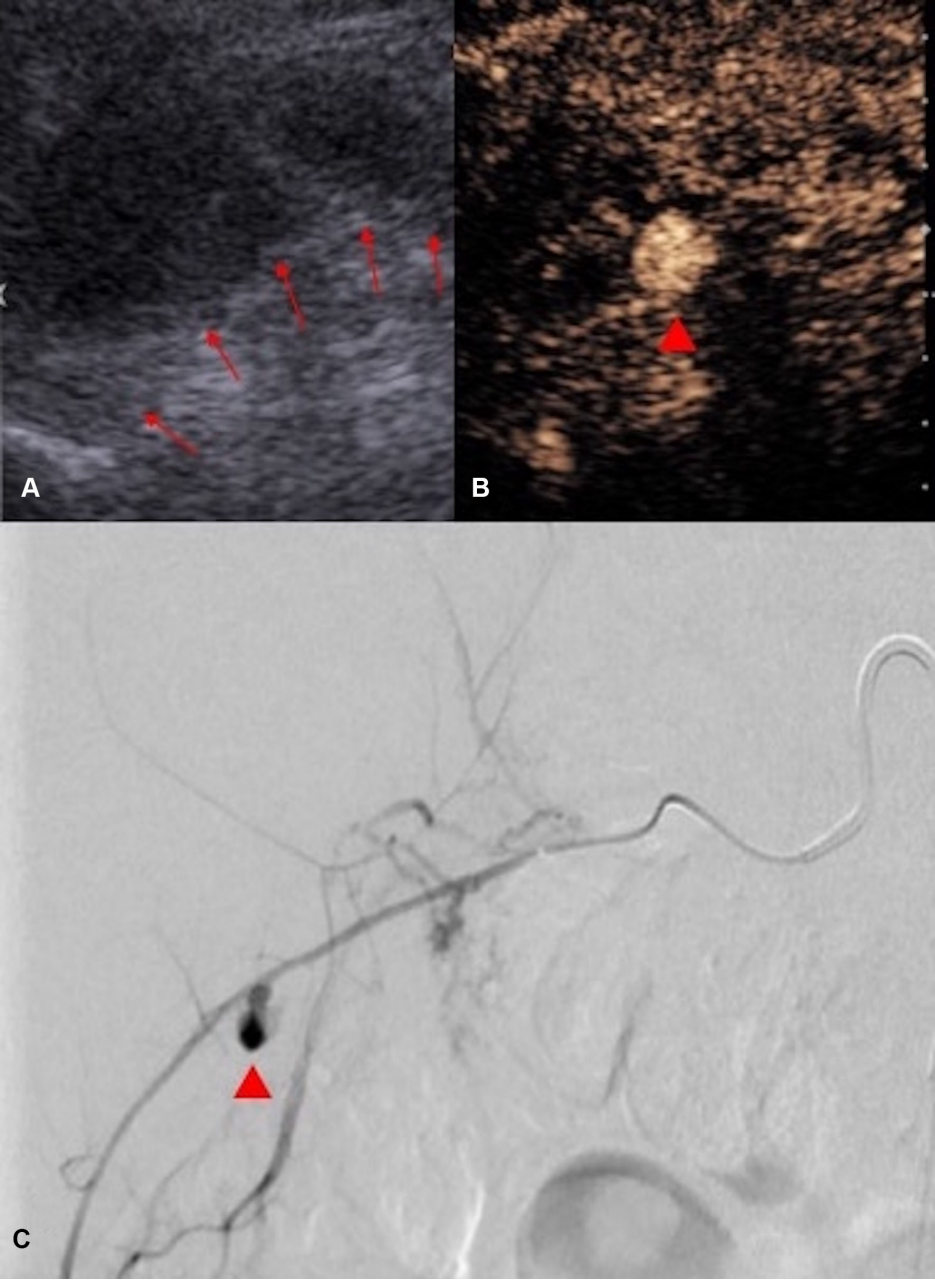
**A** Grayscale US image following a renal biopsy demonstrated a perinephric hematoma (boundary marked by red arrows), initially thought to be related to bleeding from the kidney. However, the corresponding CEUS (**B**) clearly demonstrates the pseudoaneurysm (red arrow head) is within the perinephric hematoma rather than within the kidney. **c** In view of the CEUS appearances, selective angiography of the right intercostal arteries was performed which indeed demonstrated a pseudoaneurysm (red arrow head) arising from a right intercostal artery. No renal arterial injury was demonstrated on angiography. CEUS in this case suggested the possible anatomic site of complicating arterial injury relating to renal biopsy and provided guidance for the subsequent embolization procedure

Extravasation of intravenous contrast medium is a well-known angiographic and CT finding of active bleeding. Conventional US imaging is able to recognize clots and hematoma but cannot determine whether intra-abdominal bleeding has spontaneously stopped or is still ongoing [73]. Active hemorrhage could be identified on CEUS as extravasation of microbubbles [74] paralleling the known CT and angiographic appearances. CEUS offers additional benefit of the capability to scan the region of interest continuously without radiation burden, allowing exclusion of the theoretical risk of missing a delayed extravasation. In additional to active bleeding, a pseudoaneurysm can develop following an arterial injury which can also be detected with CEUS (Fig. 15). CEUS detection of arterial injuries can also help identify the likely anatomic source of the ongoing bleeding without delay and guide the targeted surgical or angiographic treatment (Fig. 16). CEUS also represents an alternative to contrast-enhanced CT in the followup imaging of arterial injury relating abdominal intervention, arterial complication, without the need for irradiation or administration of iodinated contrast, which may be undesirable for patients with renal insufficiency [75].

## Pediatric application

US is routinely used as the imaging modality of choice for interventions in the pediatric population because of concern over medical ionizing radiation exposure of children. CEUS is a safe and potentially cost-effective imaging modality for pediatric population [76]. Pediatric CEUS-guided intervention represents a complementary technique for intervention guided by grayscale and color Doppler US. Lumason (Bracco Diagnostics; Monroe Township, NJ) has received regulatory approval for pediatric hepatic use for assessment of pediatric focal liver lesions in the United States [10, 77]. Pediatric CEUS has also been extensively in an “off-label” manner [78] for further indications [79] with promising results, such as in abdominal trauma [75] and in established endocavitary use in voiding urosonography [21]. Furthermore, CEUS-guided pediatric intervention has been reported for chest drainage [80]. The strength of CEUS-guided abdominal intervention over alternative imaging modalities described in current review would also apply to patients in the pediatric population to address specific clinical need, particularly as CEUS negates the need for ionizing radiation or general anesthesia required for alternative therapeutic approach.

## Limitations of CEUS

CEUS-guided abdominal intervention shares with conventional US-guided procedures some common causes for potential failure. First, when performing an interventional procedure, the acoustic window often needs to be larger than the window for a diagnostic US exam. Poor acoustic windows, resulting from rib shadows, respiratory movement, limited patient mobility and intervening bowel gas, may all increase procedural difficulty. Second, CEUS and US lack the panoramic properties of CT, and deep positions of abdominal organs or some retroperitoneal areas are not always adequately visualized. Moreover, as a prerequisite, CEUS-guided intervention requires adequate operator experience and expertise in interpreting diagnostic CEUS findings in addition to skills in conventional US-guided intervention. Finally, despite growing experience in the literature on CEUS-guided intervention [33], further comparative clinical trials may be required to fully validate the benefit of CEUS over other imaging modalities for a variety of abdominal interventional procedures.

## Conclusion

Contrast-enhanced US, as a natural progression from conventional US, lends itself well to abdominal interventions as it combines traditional advantages of ultrasound guidance with real-time enhancement information without significant side effects. The current state of knowledge suggests CEUS offers a valuable armamentarium for abdominal intervention, not only as a credible alternative to other imaging modalities such as CT, but has the potential to even surpass conventional alternatives and offer solutions for complex logistical or clinical challenges encountered in image-guided abdominal intervention.
